# Effectiveness of a self-management intervention with personalised genetic and lifestyle-related risk information on coronary heart disease and diabetes-related risk in type 2 diabetes (CoRDia): study protocol for a randomised controlled trial

**DOI:** 10.1186/s13063-015-1073-7

**Published:** 2015-12-02

**Authors:** Anna K. Davies, Nadine McGale, Steve E. Humphries, Shashivadan P. Hirani, Katherine E. Beaney, Dauda A. S. Bappa, John G. McCabe, Stanton P. Newman

**Affiliations:** Centre for Health Services Research, School of Health Sciences, City University London, Northampton Square, London, EC1V 0HB UK; Centre for Cardiovascular Genetics, British Heart Foundation Laboratories, Institute of Cardiovascular Science, University College London, Gower Street, London, WC1E 6BT UK; Wallace House GP Surgery, 5-11 St Andrew Street, Hertford, Hertfordshire SG14 1HZ UK

**Keywords:** RCT protocol, Diabetes, Self-management, Genetic testing, Physical activity, Healthy eating, Medication adherence, UKPDS

## Abstract

**Background:**

Many patients with type 2 diabetes fail to achieve good glycaemic control. Poor control is associated with complications including coronary heart disease (CHD). Effective self-management and engagement in health behaviours can reduce risks of complications. However, patients often struggle to adopt and maintain these behaviours. Self-management interventions have been found to be effective in improving glycaemic control. Recent developments in the field of genetics mean that patients can be given personalised information about genetic- and lifestyle-associated risk of developing CHD. Such information may increase patients’ motivation to engage in self-management. The Coronary Risk in Diabetes (CoRDia) trial will compare the effectiveness of a self-management intervention, with and without provision of personalised genetic- and lifestyle-associated risk information, with usual care, on clinical and behavioural outcomes, the cognitive predictors of behaviour, and psychological wellbeing.

**Methods/Design:**

Participants will be adults aged 25–74 years registered with general practices in the East of England, diagnosed with type 2 diabetes, with no history of heart disease, and with a glycated haemoglobin level of ≥6.45 % (47 mmol/mol). Consenting participants will be randomised to one of three arms: usual care control, group self-management only, group self-management plus personalised genetic- and lifestyle-associated risk information. The self-management groups will receive four weekly 2-hour group sessions, focusing on knowledge and information sharing, problem solving, goal setting and action planning to promote medication adherence, healthy eating, and physical activity. Primary outcomes are glycaemic control and CHD risk. Clinical data will be collected from GP records, including HbA1c, weight, body mass index, blood pressure, and HDL and total cholesterol. Self-reported health behaviours, including medication adherence, healthy eating and physical activity, and cognitive outcomes will be assessed by questionnaire. Measures will be taken at baseline, 3 months (questionnaire only), 6 months and 12 months post-baseline.

**Discussion:**

This study will determine whether the addition of personalised genetic- and lifestyle-associated CHD risk information to a group self-management intervention improves diabetes control and CHD risk compared with group self-management and usual care. Effectiveness of the combined intervention on health behaviours cognitions theorised to predict them, and psychological outcomes will also be investigated.

**Trial registration:**

This study has been registered at ClinicalTrials.gov; registration identifier NCT01891786, registered 28 June 2013.

**Electronic supplementary material:**

The online version of this article (doi:10.1186/s13063-015-1073-7) contains supplementary material, which is available to authorized users.

## Background

Approximately 2.3 million people in the UK aged over 17 years have diagnosed diabetes, of which 90 % are diagnosed with type 2 [1]. This number is expected to rise to 4 million by 2025 [2]. Complications in diabetes arise from poor glycaemic control [3], and include renal disease, retinopathy, and peripheral vascular disease [1]. People with diabetes have two times the risk of developing coronary heart disease (CHD) compared with the general population [1], and CHD is the cause of death in 52 % of people with diabetes [4]. Good glycaemic control has, however, been found to be associated with reductions in the complications associated with diabetes and the risk of developing CHD [3].

Glycaemic control and the health risks associated with diabetes can be managed through appropriate self-management behaviours including taking medication appropriately, regular physical activity, following a healthy diet and checking feet. These behaviours are also established as important in the prevention of CHD risk in people with and without diabetes. Therefore, interventions to increase adherence to these behaviours in patients with type 2 diabetes will have the dual benefit of reducing both the risk of diabetes complications and CHD.

Many people with diabetes experience difficulties in consistently carrying out the recommended behaviours. To support patients with diabetes to achieve better glycaemic control, the Quality and Outcomes Framework (QOF) recommends that all patients with diabetes are referred to group educational intervention within 9 months of diagnosis [5]. In recognition that the provision of information is insufficient to achieve the required behaviour change, self-management interventions (SMIs) have been developed and recognised as a key part of the delivery of care for people with diabetes by the National Institute for Health and Care Excellence (NICE) [6, 7].

SMIs for people with diabetes have been widely implemented both in the UK and elsewhere. Systematic and meta-analytic reviews indicate that they are effective in improving glycaemic control (glycated haemoglobin (HbA1c) [8, 9]). However, few studies have investigated the effect of SMIs on other indicators of CHD risk, with the exception of body mass index (BMI) [9]. A review of systematic reviews of diabetes SMIs found that fewer than half of included studies investigated outcomes associated with CHD risk, such as blood pressure and lipid profile [10], thus investigation of intervention effects on clinical risk factors associated with CHD is warranted.

Reviews of the effect of SMIs for diabetes have indicated that the most effective interventions are those that target specific disease prevention behaviours such as healthy eating and physical activity [9], and those that use theory to specify the intervention components [11]. A systematic review has indicated that interventions employing techniques from social learning theory (SLT) [12, 13] such as modelling, increasing self-efficacy and skills rehearsal were most effective for changing health behaviours [11]. The University College London Diabetes Self-Management Programme (UCL-DSMP) [14] is one such programme, based on SLT and employing these techniques. Compared with standard care, this intervention resulted in greater positive change in dietary behaviours and physical activity, and showed a trend towards significance in predicting greater improvements in HbA1c after 3 months.

Evaluations of SMI interventions can be enhanced by the measurement of their effects on the cognitive predictors of health behaviour, described in theories of behaviour. Investigating the effect of an intervention on cognitions can provide an explanation of intervention effects or null findings [15]. The Health Action Process Approach (HAPA) [16] integrates several theories describing the predictors of health behaviour, and states behavioural intention or motivation is a key determinant of it. Antecedents of intention include perceived risks, self-efficacy to perform, and expectancies about the outcomes of the behaviour. Planning, social support, and maintenance and recovery self-efficacy are specified as post-intentional cognitions that predict behaviour. The HAPA has been found to explain variance in intentions and behaviour for several behaviours including healthy eating in a healthy population [17], and physical activity in patients with heart failure [18].

### Motivating engagement in self-management interventions: personalised risk estimates

Increasing motivation to engage in and maintain self-management behaviours may be one way to increase the effect of SMIs on glycaemic control and engagement in healthy behaviours. Current QOF guidance requires that patients receive information about their lifestyle-associated risk of CHD annually, using standardised risk calculators e.g. the UK Prospective Diabetes Study (UKPDS) [19]. It has been put forward that the salience of this information about this modifiable risk may be increased by providing it alongside information about unmodifiable genetic risk of developing CHD [20].

Recent developments in the field of genetics have provided the opportunity to investigate the behavioural and clinical effect of providing a personalised estimate of both genetic- and lifestyle-associated risk of CHD. A CHD genetic risk score based on 19 CHD risk loci has been evaluated and found to have potential clinical utility in addition to conventional risk factors in a group of UK middle-aged healthy men [21]. This genetic risk score can then be combined with a conventional risk score from lifestyle risk calculators to give an overall estimate of 10-year CHD risk.

Currently there is no evidence to suggest individuals will perceive engaging in risk-reducing behaviours to be less beneficial should they receive a high risk result [22], and evidence indicates that participants are not likely to perceive genetics to be the sole cause of illness [23]. However, there is limited evidence that provision of personalised risk information alone will effect behaviour change where support to change behaviour is not provided. A Cochrane Review [24] has identified a small number of clinical trials in which behavioural effects of genetic risk information have been examined, and found no effect of receiving a test result on smoking cessation, physical activity, taking medication and vitamin use. An effect on dietary changes was found in only one study. The review also found that while they aimed to change behaviour, these studies did not report the use of additional behaviour change techniques such as goal setting or coping planning. However, there is some evidence for an effect of receiving a genetic risk result on the cognitions that predict behaviour; an effect of receiving a test result on smoking cessation intentions was found for analogue studies in which behavioural outcomes were not investigated due to the absence of an available test. Furthermore, receiving a risk result has been found to predict change in other cognitions theorised to predict behaviour in clinical and analogue studies, including fear arousal, perceptions of risk of developing disease, and perceived effectiveness of the target behaviour in reducing risk. Further intervention may therefore be required to help translate these cognitive changes into behaviour change. Together these findings underline the potential utility of investigating the behavioural and clinical effects of providing personalised genetic- and lifestyle-associated risk information in conjunction with an SMI.

### Study aims

The Coronary Risk in Diabetes study (CoRDia) aims to investigate the effect of an SMI with and without personalised genetic- and lifestyle-associated risk information, compared with usual care. The primary outcomes are glycaemic control measured as HbA1c, and percentage CHD risk assessed using the UKPDS risk calculator.

Secondary outcomes are other indicators of CHD risk including total and high-density lipoprotein (HDL) cholesterol, blood pressure, weight and BMI, and behaviours including adherence to medication, diet and physical activity. The effect of personalised risk information and the SMI on the theory-based cognitive predictors of behaviour, quality of life, psychological wellbeing, and emotional response to having a genetic test will also be examined.

## Methods/Design

A three-arm randomised controlled trial (RCT). Eligible participants will be randomly allocated to either a group self-management intervention (SMI), SMI plus risk results (SMI + RR), or standard usual care. See Additional file 1 for the complete SPIRIT checklist.

### Setting

The study will be conducted in GP surgeries and community diabetes clinics within the National Institute for Health Research (NIHR) Eastern Clinical Research Network (CRN). To maximise patient participation in this study two models of recruitment and intervention delivery are proposed (i) standalone and (ii) hub and spoke. Standalone practices will recruit participants from their own patient lists and deliver the interventions in-house. In the hub and spoke model, one practice will act as the hub taking responsibility for recruitment and intervention delivery for smaller local spoke practices that identify eligible patients from their patient lists.

### Ethical approval and trial registration

Ethical approval for this study has been granted by the East of England Research Ethics Committee (ref 12/EE/0437). Local governance and assurances were issued by Norfolk and Suffolk Primary and Community Care Research Office, Cambridgeshire Community Services National Health Service (NHS) Trust, and Essex and Hertfordshire Comprehensive Local Research Network (CLRN) Office. A Data Monitoring Committee will be convened to review interim data and monitor safety and overall conduct of the trial. This study has been registered at ClinicalTrials.gov; registration identifier NCT01891786.

### Recruitment

#### Primary care providers

The Eastern CRN will recruit suitable primary care providers. Participating GP surgeries or community diabetes clinics must be research active and demonstrate adequate numbers of eligible patients. Recruiting staff will need to have received Good Clinical Practice (GCP) training. Practices will be offered CRN research nurse support where they do not have sufficient resources to complete all study activities.

#### Patients

Patients diagnosed with type 2 diabetes who are registered at a GP surgery within the East of England, and who are due an annual diabetes review within the recruitment window will be invited to participate. Eligible patients must be: aged between 25 and 74 years at the time of recruitment, of White, Afro-Caribbean or Asian-Indian ethnicity, be fluent in spoken English, and have had an HbA1c of ≥6.45 % (47 mmol/mol) in the 6 months preceding recruitment. Patients will be excluded if they have: history or diagnosis of ischaemic heart disease, transient ischaemic attack (TIA) or peripheral vascular disease, current serious or enduring mental health problems that would prevent study participation, are currently undergoing treatment for a life-threatening condition or are in the terminal stages of a condition. Participants who do not fit the ethnicity requirements will be excluded; this is because the genetic test and a primary outcome measure (UKPDS Risk Engine) have not been validated outside of the described ethnic groups. Adults who cannot consent for themselves will also be excluded.

Patients due their annual diabetes review within the 8-week recruitment window will be assessed for eligibility and invited to participate in the study via letter of invitation or opportunistically at the time of an appointment. Each practice will recruit between one and three cohorts of 15 participants.

**Fig. 1:**
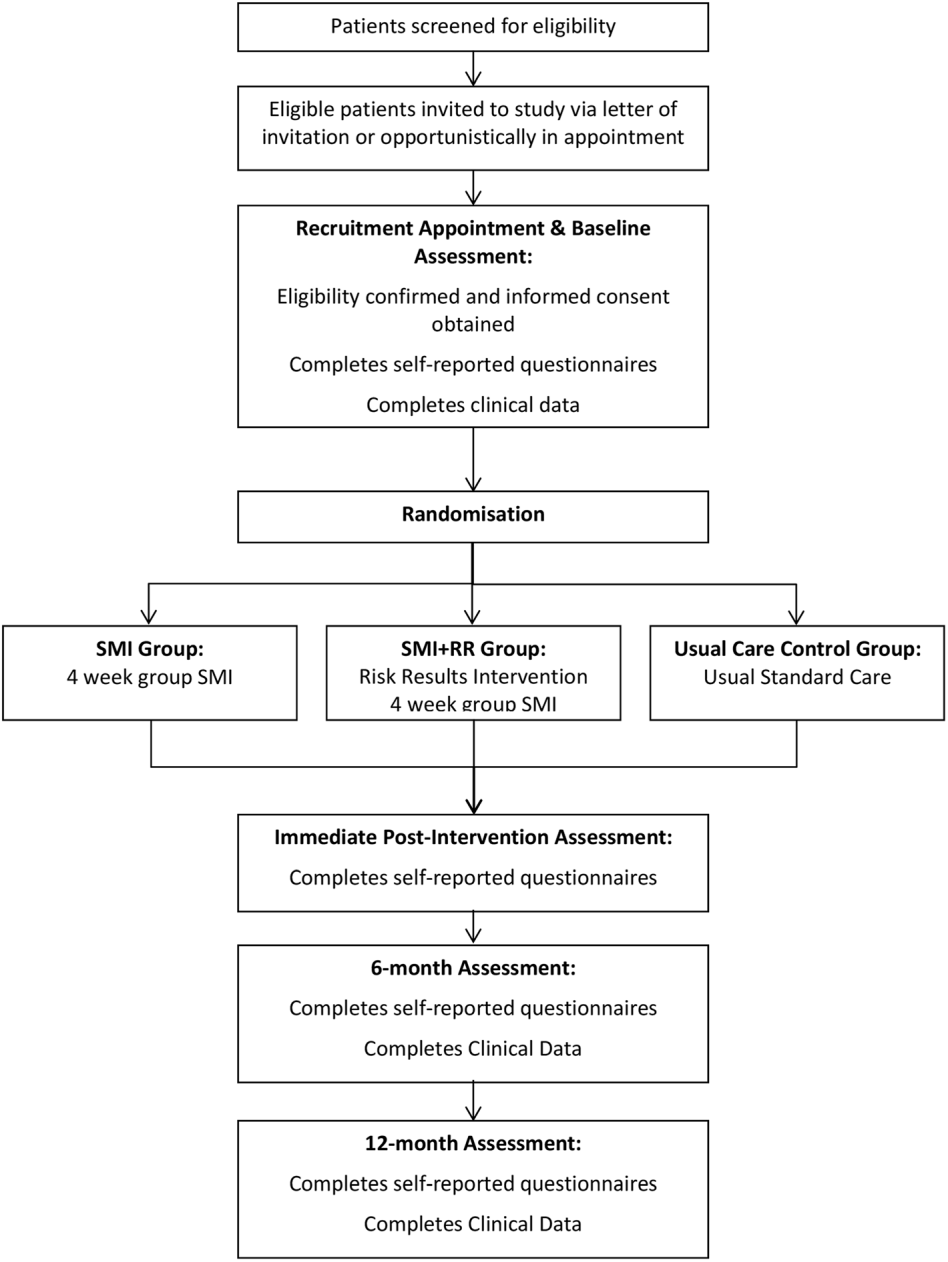
Study flow

Eligible interested patients will be invited to a consenting appointment where they will have the opportunity to review the participant information sheet with the practice or research nurse and give written informed consent to participate in the study. Once participants have completed baseline questionnaires they will be randomised to study condition, see Fig. 1.

### Randomisation and blinding

Participants will be randomised at the individual level using randomly permuted blocks. The randomisation sequence will be generated using a randomisation website [25]. Blocks of 15 allocations will be generated with a 1:1:1 ratio of study arm allocations to ensure a balance of five participants per study arm for each cohort. Each cohort of 15 participants will comprise patients recruited from a single GP practice site, or a single hub and spoke site grouping. Randomisation allocation will be concealed in consecutively numbered opaque sealed envelopes. As with all studies of this type it is not possible to blind participants, the recruiting nurse, or intervention facilitators to group allocation once the envelope has been opened. The data will be analysed by a researcher blinded to the allocation of the groups.

### Study interventions

#### Self-management intervention

Participants allocated to the SMI group and the SMI + RR group will attend group self-management sessions for 4 consecutive weeks, which will be held at a local GP surgery or community practice, in addition to their usual care. Each session will last approximately 2 hours, and will be facilitated by an SMI-trained practice or research nurse with experience of providing clinical care to patients with diabetes, paired with a health psychologist researcher or layperson. Ten participants will attend each SMI group (five SMI only, five SMI + RR). The facilitator will keep an attendance log. Intervention sessions will be audio recorded where participants provide permission, to investigate intervention fidelity.

All SMI facilitators will attend a 2-day self-management training course led by two experienced self-management practitioners and trainers. The training comprises teaching regarding theories of behaviour change and self-management. Facilitators will be taught how to use the SMI manual and will practice skills for managing groups. After training they will be supported to deliver the intervention through pre- and post-session briefing meetings each week within their facilitator pairings, and with the research team.

The SMI will be adapted from the UCL-DSMP [14]. See Additional file 2 for sample excerpts from the CoRDia SMI facilitator manual. For three of the four sessions the group will address specific target behaviours: medication adherence, healthy eating and physical activity respectively. For the fourth session, participants will be invited to identify additional target behaviours that they believe to be important in managing diabetes and/or CHD risk (e.g. foot care, smoking cessation). The following behaviour change techniques [26] will be delivered in each session:

1. **Knowledge and information sharing**: Each session will include brief didactic teaching to ensure that all participants have a clear understanding of the importance of, and how to manage, their diabetes in relation to the target behaviour.
2. **Problem solving**: Participants will be asked to brainstorm difficulties that they experience with \carrying out the target behaviour. Difficulties that several members of the group are experiencing, or would like to discuss, will be selected and strategies to overcome them elicited from the group. Strategies for several difficulties can be discussed, subject to the requirements of the group.
3. **Action planning**: In the first session, the principles of goal setting and action planning will be didactically presented. At the end of each session participants will be invited to set a goal and develop a detailed written action plan for the following week, targeting the behaviour in question. The action plan includes information about the target behaviour, the frequency and intensity with which it will be carried out, and any plans they need to make to overcome anticipated difficulties. Participants will rate their confidence to carry out the behaviour on a scale of 1–10. Where participants wish to (e.g. if they do not have difficulties with taking medication), an alternative health behaviour can be targeted. The facilitator will provide support to write the action plan and then will model reporting their personal action plan to the group. The group will be invited to report their own.
4. **Goal review and praise/reward**: For the second and subsequent sessions, goal review is carried out, where participants report back to the group about their goal attempt. Where a goal has been partially achieved or not achieved, strategies to overcome experienced problems will be briefly brainstormed by the group. Participants who have been successful or partially successful in achieving their goal will be praised by the facilitator and encouraged to recognise their success.

#### Risk results intervention

Participants allocated to the SMI + RR group will be asked to review and sign the consent form specific to the genetic test and provide a saliva sample for analysis using the Oragene DNA Self-collection Kit (DNA Genotek Inc., Ontario, Canada). Deoxyribonucleic acid (DNA) will be extracted according to the manufacturer’s instructions and analysed as described below. Participants will be invited to attend a one-on-one appointment with the researcher in the week prior to the first SMI group session, where a trained researcher will present the results of the risk report to each participant using a script to ensure consistency in delivery.

#### The risk report

Risk reports will be personalised to describe individual CHD risk, defined as risk of having a myocardial infarction (MI). There are three sections to the risk report, detailing (i) genetic lifetime CHD risk presented as a graph, with the patient’s personal risk relative to average risk, (ii) 10-year lifestyle CHD risk presented as a graph, reporting absolute and relative risk to the average person’s percentage risk. This is supported by text detailing the participant’s estimated lifestyle risk, the risk for the average person of their age, ethnic group and gender, and risk relative to the average person, expressed as a percentage. (iii) Combined genetic and lifestyle overall 10-year CHD risk presented in the same manner as lifestyle risk. The report will state that it is possible to reduce lifestyle risk by engaging in health behaviours e.g., eating more healthily and becoming more physically active, all of which are addressed in the SMI. Participants will be given their printed personalised risk report to keep for their own records; copies of individual’s risk reports will not be stored or made available to their care provider.

#### Genetic analysis and risk calculation

Genotypes for 19 CHD risk single nucleotide polymorphisms (SNPs) will be determined using the Randox Cardiac Risk Prediction Array (Randox Laboratories Ltd., Crumlin, UK). In the event of any discrepancies, genotypes will be confirmed by Sanger sequencing. The number of risk alleles for each SNP carried by an individual will be multiplied by its reported meta-analysis effect size for CHD, for method see [21] to give an individual’s genetic risk score. To put the genetic risk score into context, the individual genetic risk score will then be adjusted for the population average genetic risk score. The population average risk is calculated by multiplying the expected frequency of the three genotypes (based on the allele frequency determined in the source publications, see [21] for each SNP by the number of risk alleles corresponding to that genotype). These products are then summed for all the SNPs. The comparative average person is someone with type 2 diabetes of the same age, ethnicity and gender as the individual participant, with the average systolic blood pressure (BP) and lipid ratio determined using data from the University College Diabetes and Cardiovascular Study (UDACS) [27]; data presented in Additional file 3. An appropriate value for HbA1c has been set on advice from a group clinician. The 10-year lifestyle CHD risk will be estimated using the UKPDS risk engine v2.0 [28]. To give the combined 10-year CHD risk incorporating both lifestyle and genetic risk the population-adjusted genetic risk will be then added to the UKPDS risk.

### Usual care control group

Those randomised to usual care will be advised to continue to manage their diabetes as they would normally. Usual care will be documented across practices and community services involved in the study using a checklist informed by the General Medical Services QOF 2014/15 and NICE clinical guidelines for the management of type 2 diabetes (modified March 2014). The checklist will be developed in consultation with specialist primary care diabetes staff.

### Measures

#### Socio-demographic information

Age, ethnicity, marital status, and education and employment status will be collected at baseline. Participants will also be asked to self-report whether they have attended any health management programmes previously and to indicate if there is a known history of CHD in their immediate family.

### Primary outcomes

*HbA1c.* Change in blood glucose control as measured by glycated haemoglobin (HbA1c) will be recorded from patient’s medical records. HbA1c reflects average plasma glucose concentration over an 8–12-week period and will be collected at baseline and 6 and 12 months post-baseline.

*UKPDS risk.* Ten-year risk of CHD defined as fatal, and non-fatal, MI will also be calculated at baseline and 6 and 12 months post-baseline using the UKPDS risk engine (v2.0) [28]. UKPDS is a risk calculator specifically for people with type 2 diabetes. Percentage risk is calculated from participants’ age, years since diabetes diagnosis, presence of atrial fibrillation, reported smoking behaviour, sex, ethnicity, HbA1c, systolic BP, and total and HDL cholesterol.

### Secondary outcomes

#### Clinical

The following clinical measures will also be recorded from participant’s medical records at baseline, and 6 and 12 months post-baseline to be considered independently: (i) sitting systolic and diastolic BP, (ii) total cholesterol, (iii) HDL cholesterol, and (iv) BMI.

If available in patient records, the following additional measures will also be recorded: (i) smoking status, (ii) blood glucose fasting sample, (iii) low-density lipoprotein (LDL) cholesterol, (iv) triglycerides, (v) serum creatinine, (vi) estimated glomerular filtration rate (eGFR), (vii) urine albumin to creatinine ratio (urine ACR), (viii) foot risk assessment, and (ix) impotence.

The following self-reported behavioural, cognitive and psychological measures will be measured at baseline, immediately post-intervention (approximately 3 months post-baseline), and 6 months and 12 months post-baseline.

#### Behavioural

*The summaries of self-care activities measure* will assess diabetes self-management behaviours for diet, exercise, blood sugar testing, smoking and diabetes medications [29].

#### Cognitive predictors of health behaviours

The cognitive predictors of behaviour described in the HAPA will be assessed using measures adapted from those used for the Risk Appraisal Consequences in Korea Study [30]. Changes to wording have been made to increase clarity in English. Measures assess:

1. *Perceived risk of developing heart disease*: The following three scales assess perceived risk in relation to six types of CHD: high cholesterol, heart attack, high blood pressure, stroke, angina, heart disease. Participants respond on a 7-point Likert scale for each item.
  - *Absolute risk* assessing own perceived risk.
  - *Absolute risk for average person with diabetes* assessing perceived risk of average person of same age, gender, ethnic group.
  - *Comparative risk of developing CHD* assessing perceived risk in comparison with average person with diabetes.
2. *Perceived severity of CHD*: assessed for each of the six types of CHD on a 7-point Likert scale for (i) average person, and (ii) self.
3. *Intentions to engage in each of the five target behaviours*: healthy eating, physical activity, checking blood sugars (where prescribed), taking medication, stopping smoking. Participants respond on a 4-point Likert scale for each behaviour.
4. *Planning with respect to five target behaviours*: five scales with up to 10 items assess plans for when, where, what to do, how often and plans needed to engage in each of the target health behaviours. Responses are given on a 4-point Likert scale.
5. *Outcome expectancies towards each of target behaviours*: assessed on five scales comprising 6–12 items, relating to potential positive and negative outcomes of the target behaviour. Participants respond on a 4-point Likert scale.
6. *Self-efficacy to perform each of the five target behaviours*:
  - *Confidence to perform the behaviour regularly*: five items relating to target behaviours. Participants respond on a 10-point Likert scale.
  - *Confidence to overcome barriers to target behaviour*: five scales relating to each target behaviour. Each scale has 10–13 items relating to problems that may be encountered.

Participants respond on a 10-point Likert scale.

### Psychological outcomes

*Depression and anxiety* will be assessed using the Hospital Anxiety and Depression Scale (HADS) [31] a 14-item questionnaire for use in patients with physical health problems. Total scores on each subscale are summed (score range = 0–21), with higher scores indicating greater levels of anxiety or depression. The HADS has demonstrated good reliability and validity among primary care patients [32].

*Health-related quality of life* will be assessed using the Short-Form Health Survey (version 2) (SF-12v2), which comprises two subscales: The Mental Component Score (MCS) and the Physical Component Score (PCS), reflecting mental and physical health-related quality of life respectively. Total scores range from 0–100 with higher scores indicating greater health status. Both subscales of the SF-12v2 have reported good reliability and validity [33].

### Response to risk results

For those in the SMI + RR group, participant responses to receiving personalised risk information will be assessed immediately post-intervention, and 6 and 12 months post-baseline.

*Recall and comprehension of risk results*: Questions will be developed for this study. Participants will be asked to recall from memory their absolute lifestyle and overall risk percentage, and to identify the description relative to the average person of their genetic risk result from a list of six statements. Comprehension of the genetic test result will further be assessed by asking participants to identify whether four descriptive statements about their result are true or false, e.g., ‘*Your genetic test result is based on the number of gene variants you have*’*.*

*Psychological impact of receiving a genetic and lifestyle risk result*: Will be assessed by adapting the Impact of Genetic Testing for Alzheimer’s disease (IGT-AD) scale [34] to make it appropriate to CHD risk. This measure comprises distress and positive experiences subscales; three questions will be eliminated from the distress subscale as they are not relevant to this study. Responses to this adapted 13-item measure will be recorded on a 5-point Likert scale, with higher scores indicating greater psychological distress; responses to the positive experiences subscale are reverse scored.

### Power calculation

A clinically important change in HbA1c level of 12.5 % (SD 15.5 %) was identified from a Health Technology Assessment of blood glucose monitoring devices [35] over a period of 18 months. This represents a reduction in HbA1c (%) from 8 to 7 or from 9 to 7.9. As the current study has a shorter follow-up, we base our sample size calculations on a conservative change that would be clinically achievable in this time frame: an HbA1c change of 6.25 % representing a reduction from an HbA1c (%) of 8 to 7.5 or from 9 to 8.44 (this represents a change from 63.9 mmol/ml to 59.9 mmol/ml; and 74.9 mmol/ml to 69.4 mmol/ml).

The expected 6.25 % change in HbA1c (SD = 15 %) represents an effect size of d = 0.40, a small to moderate effect size (d = 0.2 – small, 0.5 – medium and 0.8 – large effect); equivalent to effect size f = 0.2 in G*Power [36] calculated for repeated measures, between-subjects analysis of variance (ANOVA) analyses. Based on the current design (three groups, four measurement points), alpha = 0.05, power = 80 %, f = 0.20 and a correlation between measurement points of 0.70, G*Power indicates a total sample size of 192. With an expected attrition rate of 20 %, a sample size of 240 (80 per study arm) will be sought.

### Analysis

#### Preliminary analyses

Descriptive statistics will be used to assess levels of missing data, the frequency of events, participant characteristics, and the central tendency and dispersion of key variables. If missing data analyses indicate large levels of missing data (>10 % on any variable), multiple imputation using SPSS Markov Chain Monte Carlo procedures (IBM Corp., Armonk, NY, USA) will be implemented to produce 10 imputed datasets. These will each be analysed as specified below; thereafter standard multiple imputation procedures will be used to combine the multiple scalar and multivariate estimates quantities. If missing data levels are lower, a single imputation procedure will be implemented, followed by standard analyses procedures.

#### Main analyses

Spearman’s or Pearson’s correlations will determine relationships between variables and chi-squared analyses will be used to compare rates data. For the main group differences and time-based analyses, data will be analysed using intention-to-treat principle and then repeated with per protocol subsamples, for sensitivity analyses. For the main continuous outcome measures (HbA1c and CHD risk data) and secondary outcomes 3 x 3 mixed analysis of covariance (ANCOVA) controlling for existing group differences on key process variables will be conducted for each outcome, with post hoc comparisons using Sidak’s correction. Should the data meet the criteria for multi-level modelling, these analyses will be conducted in lieu of ANCOVA.

## Discussion

Patients with type 2 diabetes frequently require support to self-manage health behaviours such as medication adherence, consuming a healthy diet, and engaging in physical activity, which are known to reduce the risks associated with poor glycaemic control in diabetes, including coronary heart disease. Motivation to engage in, and the effectiveness of, a self-management intervention may be increased by providing estimates of genetic- and lifestyle-associated risk of CHD. This study will use a novel genetic test to test the effects of an SMI with and without personalised genetic- and lifestyle-associated risk estimates on clinical indicators of diabetes and CHD risk, health behaviours, cognitive predictors of behaviour and psychological outcomes in a cohort of patients with type 2 diabetes.

## Trial status

Patient recruitment has been ongoing since December 2013 and is expected to end June 2015. Study completion is estimated to be June 2016.

## Additional files

Additional file 1:
**SPIRIT checklist CoRDia protocol.** Completed SPIRIT checklist relevant to the study protocol. (DOCX 59 kb) Additional file 2:
**Sample excerpts from the CoRDia SMI.** Selected sample excerpts from the CoRDia SMI Facilitator Manual including introductory material, problem solving, and action planning sections. (PDF 905 kb) Additional file 3:
**UDACS average person risk data.** CoRDia protocol. Data from the University College Diabetes and Cardiovascular Study (UDACS): comparator average person risk factor data. Average person risk factor data from the University College Diabetes and Cardiovascular Study (UDACS) used to determine average person comparator in intervention coronary heart disease risk reports. (DOCX 22 kb)

## Abbreviations

BMI: Body Mass Index
BP: Blood Pressure
CHD: Coronary Heart Disease
CLRN: Comprehensive Local Research Network
CoRDia: Coronary Risk in Diabetes
CRN: Clinical Research Network
DNA: Deoxyribonucleic acid
eGFR: estimated Glomerular Filtration Rate
GCP: Good Clinical Practice
HADS: Hospital Anxiety and Depression Scale
HAPA: Health Action Process Approach
HbA1c: Glycated haemoglobin
HDL: High-density lipoprotein
IGT-AD: Impact of Genetic Testing for Alzheimer’s disease
LDL: Low-density lipoprotein
MCS: Mental Component Scale
MI: myocardial infarction
NHS: National Health Service
HNICE: National Institute for Health and Care Excellence
NIHR: National Institute Health Research
PCS: Physical Component Scale
QOF: Quality and Outcomes Framework
RCT: Randomised controlled trial
SF-12: Short-Form Health Survey
SLT: Social learning theory
SMI: Self-management intervention
SMI group: Self-management intervention group
SMI + RR group: Self-management intervention and risk results group
SNP: Single nucleotide polymorphism
TIA: Transient ischaemic attack
UDACS: University College Diabetes and Cardiovascular Study
UCL-DSMP: University College London Diabetes Self-Management Programme
UKPDS: UK Prospective Diabetes Study
Urine ACR: Urine albumin to creatinine ratio

## References

[1] Diabetes UK. Diabetes facts and stats. 2014. https://www.diabetes.org.uk/Documents/About%20Us/Statistics/Diabetes-key-stats-guidelines-April2014.pdf. Accessed 01 June 2015

[2] Wild S, Roglic G, Green A, Sicree R, King H (2004). Global prevalence of diabetes: estimates for the year 2000 and projections for 2030. Diabetes Care..

[3] The Diabetes Control and Complications Trial Research Group (1993). The effect of intensive treatment of diabetes on the development and progression of long-term complications in insulin-dependent diabetes mellitus. N Engl J Med.

[4] Morrish NJ, Wang SL, Stevens LK, Fuller JH, Keen H (2001). Mortality and causes of death in the WHO multinational study of vascular disease in diabetes. Diabetologia..

[5] National Institute for Health and Care Excellence. Quality Outcomes Framework for Diabetes Mellitus. Guideline NM27. 2011. http://www.nhsemployers.org/~/media/Employers/Documents/Primary%20care%20contracts/QOF/2015%20-%2016/2015%2016%20QOF%20guidance%20for%20stakeholders.pdf. Accessed 01 June 2015.

[6] National Institute for Health and Care Excellence. Type 2 diabetes: the management of type 2 diabetes: NICE clinical guideline 87. NICE. 2009. http://www.nice.org.uk/guidance/cg87. Accessed 25 May 2015.

[7] Newman S, Steed L, Mulligan K (2004). Self-management interventions for chronic illness. Lancet..

[8] Norris SL, Lau J, Smith SJ, Schmid CH, Engelgau MM (2002). Self-management education for adults with type 2 diabetes: a meta-analysis of the effect on glycemic control. Diabetes Care..

[9] Heinrich E, Schaper NC, de Vries NK (2010). Self-management interventions for type 2 diabetes: a systematic review. Eur Diabetes Nurs..

[10] Worswick J, Wayne SC, Bennett R, Fiander M, Mayhew A, Weir MC (2013). Improving quality of care for persons with diabetes: an overview of systematic reviews - what does the evidence tell us?. Syst Rev..

[11] Padgett D, Mumford E, Hynes M, Carter R (1988). Meta-analysis of the effects of educational and psychosocial interventions on management of diabetes mellitus. J Clin Epidemiol..

[12] Bandura A, Adams NE, Beyer J (1977). Cognitive processes mediating behavioral change. J Pers Soc Psychol..

[13] Bandura A (1977). Self-efficacy: toward a unifying theory of behavioral change. Psychol Rev..

[14] Steed L, Lankester J, Barnard M, Earle K, Hurel S, Newman S (2005). Evaluation of the UCL diabetes self-management programme (UCL-DSMP): a randomized controlled trial. J Health Psychol..

[15] Michie S (2004). Interventions to change health behaviours: evidence-based or evidence-inspired?. Psychol Health..

[16] Schwarzer R (1992). Self-efficacy: thought control of action.

[17] Schwarzer R, Renner B (2000). Social-cognitive predictors of health behavior: action self-efficacy and coping self-efficacy. Health Psychol..

[18] Sniehotta FF, Scholz U, Schwarzer R (2006). Action plans and coping plans for physical exercise: a longitudinal intervention study in cardiac rehabilitation. Br J Health Psychol..

[19] UK Prospective Diabetes Study (UKPDS) VIII (1991). Study design, progress and performance. Diabetologia.

[20] Tercyak KP, O’Neill SC, Roter DL, McBride CM (2012). Bridging the communication divide: a role for health psychology in the genomic era. Prof Psychol Res Pr..

[21] Beaney KE, Cooper JA, Shahid SU, Ahmed W, Qamar R, Drenos F (2015). Clinical utility of a coronary heart disease risk prediction gene score in UK healthy middle-aged men and in the Pakistani population. PLoS One..

[22] Collins RE, Wright AJ, Marteau TM (2011). Impact of communicating personalized genetic risk information on perceived control over the risk: a systematic review. Genet Med..

[23] Kaphingst KA, McBride CM, Wade C, Alford SH, Reid R, Larson E (2012). Patients’ understanding of and responses to multiplex genetic susceptibility test results. Genet Med..

[24] Marteau TM, French DP, Griffin SJ, Prevost AT, Sutton S, Watkinson C (2010). Effects of communicating DNA-based disease risk estimates on risk-reducing behaviours. Cochrane Database Syst Rev..

[25] Dallal, GE. http://www.jerrydallal.com/random/assign.htm. 2008. Accessed 04 Jan 2014.

[26] Michie S, Richardson M, Johnston M, Abraham C, Francis J, Hardeman W (2013). The behavior change technique taxonomy (v1) of 93 hierarchically clustered techniques: building an international consensus for the reporting of behavior change interventions. Ann Behav Med..

[27] Dhamrait SS, Stephens JW, Cooper JA, Acharya J, Mani AR, Moore K (2004). Cardiovascular risk in healthy men and markers of oxidative stress in diabetic men are associated with common variation in the gene for uncoupling protein 2. Eur Heart J..

[28] Stevens RJ, Kothari V, Adler AI, Stratton IM (2001). The UKPDS risk engine: a model for the risk of coronary heart disease in Type II diabetes (UKPDS 56). Clin Sci (Lond).

[29] Toobert DJ, Hampson SE, Glasgow RE (2000). The summary of diabetes self-care activities measure: results from 7 studies and a revised scale. Diabetes Care..

[30] Renner B, Schwarzer R. Risk and health behaviors. Documentation of the scales of the research project: “Risk appraisal consequences in Korea” (RACK). http://userpage.fu-berlin.de/gesund/RACK-English.pdf. Berlin FU. 2003.

[31] Zigmond AS, Snaith RP (1983). The hospital anxiety and depression scale. Acta Psychiatr Scand..

[32] Bjelland I, Dahl AA, Haug TT, Neckelmann D (2002). The validity of the Hospital Anxiety and Depression Scale. An updated literature review. J Psychosom Res.

[33] Ware J, Kosinski M, Keller SD (1996). A 12-Item Short-Form Health Survey: construction of scales and preliminary tests of reliability and validity. Med Care..

[34] Chung WW, Chen CA, Cupples LA, Roberts JS, Hiraki SC, Nair AK (2009). A new scale measuring psychologic impact of genetic susceptibility testing for Alzheimer disease. Alzheimer Dis Assoc Disord..

[35] Newman SP, Cooke D, Casbard A, Walker S, Meredith S, Nunn A (2009). A randomised controlled trial to compare minimally invasive glucose monitoring devices with conventional monitoring in the management of insulin-treated diabetes mellitus (MITRE). Health Technol Assess..

[36] Faul F, Erdfelder E, Buchner A, Lang AG (2009). Statistical power analyses using G*Power 3.1: Tests for correlation and regression analyses. Beh Res Methods.

