# Supplementary material for: Effectiveness of a self-management intervention with personalised genetic and lifestyle-related risk information on coronary heart disease and diabetes-related risk in type 2 diabetes (CoRDia): study protocol for a randomised controlled trial
Source: Trials. 2015 Dec 2;16:547. doi: 10.1186/s13063-015-1073-7 (PMC4668706; doi:10.1186/s13063-015-1073-7)
Supplement: Additional file 2: — Sample excerpts from the CoRDia SMI. Selected sample excerpts from the CoRDia SMI Facilitator Manual including introductory material, problem solving, and action planning sections. (PDF 905 kb) [file 13063_2015_1073_MOESM2_ESM.pdf]

This document presents excerpts from the CoRDia Self-Management Intervention Facilitator Manual. Please note the CoRDia SMI Facilitator Manual is solely intended for use by those who have received training in group facilitation and behaviour change skills as considered appropriate by the authors for delivering the self-management intervention.

EXCERPT FROM INTRODUCTION SECTION:

What Does the Programme Entail?

The programme takes place over four weeks with one 2 to 2.5 hour session per week. All sessions are co-facilitated by a practice or research nurse and a researcher or layperson who have attended the training.

The style of the programme is such that the majority of the learning will occur from within the group. The programme is not designed to be didactic and to this end the nurse should act as a facilitator rather than solely an educator.

The key topics covered over the four weeks of the programme are:

Week	Topics addressed
1	What is Diabetes and Why is self-management Important both in relation to diabetes and in relation to coronary heart disease?<br>Medication and Diabetes
2	Healthy Eating and Diabetes
3	Healthy Eating and Diabetes<br>Physical Activity and Diabetes
4	Using skills learned in other parts of diabetes and coronary heart disease management

The first topic serves to introduce the concept of self-management in relation to diabetes and coronary heart disease. It aims to help participants to make the connection between the clinical indicators/predictors of diabetes and heart disease, and their lifestyles.

The subsequent four topics are all areas which are likely to require behaviour change. In addressing each type of behaviour, the following components of behaviour change are addressed:

- Knowledge and information sharing
- Challenging incorrect or unhelpful beliefs
- Problem solving techniques to overcome barriers
- Practical skills where required
- Setting and reviewing goals to facilitate behaviour change

While knowledge and practical skills are necessary for self-management they are often not sufficient for behaviour change. To obtain behaviour change one must consider the problems

people experience that get in the way of behaviour change. Problems can be either practical or psychological and once identified as a problem, can be addressed by teaching problem-solving skills. Among the techniques taught are: breaking down problems into manageable chunks and then identifying strategies to deal with each problem in turn. Behavioural strategies of goal setting, review and self-reward are also used to assist the problem solving process.

Psychological problems experienced may be even more difficult to overcome than practical problems and require consideration of an individual's belief system in relation to diabetes. Throughout the programme unhelpful or negative beliefs are challenged where appropriate. However, four beliefs are seen as central to behaviour change in diabetes and are therefore specifically addressed by the programme. Two general beliefs that are seen as fundamental to self-management are addressed. These are:

(i) That type 2 diabetes is a serious condition that requires appropriate care
(ii) That participants have an important role to play in managing their diabetes
(iii) That performing the behaviour as required will be effective in helping to control their diabetes.
(iv) That the individual has the ability to perform the behaviour as required. An individual's belief in their ability is known as self-efficacy and will be targeted for each behaviour.

By addressing problem solving and beliefs in addition to knowledge and practical skills, the diabetes self-management programme aims to empower patients to change and maintain appropriate self-management behaviours.

EXCERPT FROM PROBLEM-SOLVING SECTION

1.2. IDENTIFYING PROBLEMS WITH TAKING MEDICATION (15 mins)

AIM: Identify that not adhering to medication is a potential problem in managing diabetes and identify the problems that the group experience in taking medications

This section includes: (i) A Brainstorm to identify problems

What you will do…

BRAINSTORM

- Say that there are various reason why some people do not take their medication exactly as the doctor tells them and that you want the group to identify any problems they have with taking their medications

QUESTION: What reasons can you think of as to why you've not taken your medication as directed by the doctor or nurse?

- Use the guide below to facilitate group discussion; use probes if group report dew problems with adhering to medication

- Problems fall into 2 categories:
  - (i) Practical Problems
  - (ii) Emotional Problems

- Summarise the group discussion once you have exhausted the problems which patients experience

GUIDE for discussion:

Note: Try to divide the 2 categories of practical and emotional problems when scribing.

Heading for board	Prompts	Possible responses
Problems in taking medication as directed	Does anyone experience any side effects of their diabetes medication?	• Metformin can be associated with flatulence and diarrhoea<br>• This can be helped by taking tablets just after meals<br>• Sulphonylureas can increase appetite and weight gain and may cause hypoglycaemia.

SUMMARISE the brainstorm:

- The problems that you have mentioned as causing you difficulties with taking your medications as prescribed are: …

1.3. STRATEGIES FOR OVERCOMING PROBLEMS WITH TAKING MEDICATION (15 mins)

AIM: To identify strategies to overcome problems with taking medication

This section includes: (i) A Brainstorm to identify strategies to overcome problems

What you will do…

BRAINSTORM

- Identify a problem from the list that either you identified as a common difficulty from the previous discussion, or a problem that the group would like to look at.
- Explain that as a group you are going to look at some strategies to overcome the problems that people are experiencing

QUESTION: Looking at problem x, has anyone got any suggestions for ways to overcome this problem?

- Depending on time available try to find strategies for more than one problem by repeating the question above for a new problem
- Summarise the group discussion once you have exhausted the problems which patients experience
- Encourage the participants to consider selecting a strategy if needed
- Use the brainstorm guide below to facilitate group discussion

GUIDE for Brainstorm:

Question	Prompt	Possible responses
Problem 1: Experiencing side-effects	What strategies do some of you use to avoid e.g., side effects<br><br>Did anyone find a way to overcome this side-effect?	• When speaking to the doctor it is important to be as clear as possible about the problems you experience.<br>• It may help to make a note of exactly when and in what context the medication causes you problems.

EXCERPT FROM ACTION PLANNING SECTION

1.4. ACTION PLANNING FOR MEDICATION ADHERENCE (10 mins)

AIM: Explain the general principles behind goal-setting and action planning. Help each individual to set a specific medication adherence goal
This section includes: (i) Action Planning

What you will do…

ACTION PLANNING

- Explain to the group that they will now set a goal for how they are going to take their medication in the next week

- Explain goal-setting using SETTING GOALS POSTER, ACTION PLANS (SHORT-TERM GOALS) POSTER, and CHARACTERISTICS OF EFFECTIVE ACTION PLANNING POSTER

- Ensure the following concepts are covered:
  1. Long-term v short-term goals (action plans)
  2. Specificity of action plans
  3. Confidence in setting goals: it is important for participants to set themselves up for success and choose a goal where they feel confident
  4. Planning for overcoming problems to achieving their goals

- **Model** how an action plan should be made by setting one yourself and sharing with the group, using the ACTION PLANNING SHEET POSTER.

- Ask the participants to each make an action plan using the ACTION PLANNING SHEET HANDOUT

- Ask participants to report their action plan back to the group, checking they have understood the instructions

- Check the **confidence** levels and try to think of strategies to overcoming problems in achieving the goal if confidence is below 7. *"What's stopping you from feeling more confident?"* Suggest the participant plans an alternative if they are not confident in achieving the goal they have set. Alternatively, if the participant is reporting a confidence below 7 but is unwilling to change their action plan then do not make them do so.

- If a participant does not need to set a medication adherence goal (i.e., if they have discussed that they take their medication as directed) then suggest that they think about another health goal they may wish to tackle and apply the same steps
