# Supplementary material for: Effectiveness of a self-management intervention with personalised genetic and lifestyle-related risk information on coronary heart disease and diabetes-related risk in type 2 diabetes (CoRDia): study protocol for a randomised controlled trial
Source: Trials. 2015 Dec 2;16:547. doi: 10.1186/s13063-015-1073-7 (PMC4668706; doi:10.1186/s13063-015-1073-7)
Supplement: Additional file 3: — UDACS average person risk data. CoRDia protocol. Data from the University College Diabetes and Cardiovascular Study (UDACS): comparator average person risk factor data. Average person risk factor data from the University College Diabetes and Cardiovascular Study (UDACS) used to determine average person comparator in intervention coronary heart disease risk reports. (DOCX 22 kb) [file 13063_2015_1073_MOESM3_ESM.docx]

Table 1: Risk factor data for Caucasian males in UDACS.

| Age | Systolic  Blood Pressure (mmHg) | Cholesterol (mmol/l) | HDL  Cholesterol  (mmol/l) |
| --- | --- | --- | --- |
| 20 | 129.5 | 5.76 | 1.06 |
| 30 | 131.6 | 5.57 | 1.09 |
| 40 | 133.8 | 5.38 | 1.12 |
| 50 | 135.9 | 5.19 | 1.14 |
| 60 | 138.1 | 5.00 | 1.17 |
| 70 | 140.4 | 4.81 | 1.20 |
| 80 | 142.6 | 4.63 | 1.23 |

The mean value for each trait at a given age is shown.

Table 2: Risk factor data for Caucasian females in UDACS

| Age | Systolic  Blood Pressure (mmHg) | Cholesterol (mmol/l) | HDL  Cholesterol  (mmol/l) |
| --- | --- | --- | --- |
| 20 | 129.1 | 5.67 | 1.29 |
| 30 | 131.9 | 5.60 | 1.31 |
| 40 | 134.7 | 5.52 | 1.34 |
| 50 | 137.7 | 5.44 | 1.37 |
| 60 | 140.7 | 5.36 | 1.39 |
| 70 | 143.7 | 5.29 | 1.42 |
| 80 | 146.9 | 5.21 | 1.45 |

The mean value for each trait at each a age is shown.

Table 3: Risk factor data for Asian Indian males in UDACS.

| Age | Systolic  Blood Pressure (mmHg) | Cholesterol (mmol/l) | HDL  Cholesterol  (mmol/l) |
| --- | --- | --- | --- |
| 20 | 121.7 | 4.49 | 0.92 |
| 30 | 125.7 | 4.55 | 0.96 |
| 40 | 129.7 | 4.60 | 1.00 |
| 50 | 134.0 | 4.66 | 1.03 |
| 60 | 138.3 | 4.71 | 1.07 |
| 70 | 142.8 | 4.77 | 1.11 |
| 80 | 147.4 | 4.82 | 1.15 |

The mean value for each trait at each a given age is shown.

Table 4: Risk factor data for Asian Indian females in UDACS.

| Age | Systolic  Blood Pressure (mmHg) | Cholesterol (mmol/l) | HDL  Cholesterol  (mmol/l) |
| --- | --- | --- | --- |
| 20 | 111.0 | 4.38 | 0.85 |
| 30 | 117.3 | 4.62 | 0.95 |
| 40 | 123.9 | 4.86 | 1.08 |
| 50 | 130.8 | 5.11 | 1.22 |
| 60 | 138.2 | 5.35 | 1.37 |
| 70 | 146.0 | 5.59 | 1.55 |
| 80 | 154.2 | 5.84 | 1.75 |

The mean value for each trait at each a given age is shown.

Table 5: Risk factor data for Afro-Caribbean males in UDACS.

| Age | Systolic  Blood Pressure (mmHg) | Cholesterol (mmol/l) | HDL  Cholesterol  (mmol/l) |
| --- | --- | --- | --- |
| 20 | 129.2 | 6.41 | 1.19 |
| 30 | 130.9 | 6.10 | 1.25 |
| 40 | 132.7 | 5.80 | 1.31 |
| 50 | 134.4 | 5.49 | 1.38 |
| 60 | 136.2 | 5.19 | 1.45 |
| 70 | 138 | 4.89 | 1.52 |
| 80 | 139.8 | 4.58 | 1.60 |

The mean value for each trait at each a given age is shown.

Table 6: Risk factor data for Afro-Caribbean males in UDACS.

| Age | Systolic  Blood Pressure (mmHg) | Cholesterol (mmol/l) | HDL  Cholesterol  (mmol/l) |
| --- | --- | --- | --- |
| 20 | 137.5 | 4.15 | 1.78 |
| 30 | 140.6 | 4.37 | 1.71 |
| 40 | 143.8 | 4.58 | 1.64 |
| 50 | 147.1 | 4.80 | 1.58 |
| 60 | 150.4 | 5.01 | 1.52 |
| 70 | 153.8 | 5.23 | 1.46 |
| 80 | 157.3 | 5.44 | 1.40 |

The mean value for each trait at a given age is shown.
